# Concordance between influential adverse treatment outcomes and localized prostate cancer treatment decisions

**DOI:** 10.1186/s12911-022-01972-w

**Published:** 2022-08-24

**Authors:** Rachel A. Pozzar, Niya Xiong, Fangxin Hong, Christopher P. Filson, Peter Chang, Barbara Halpenny, Donna L. Berry

**Affiliations:** 1 Dana-Farber Cancer Institute, 450 Brookline Ave., Boston, MA 02215 USA; 2 Emory University, 100 Woodruff Circle, Atlanta, GA 30322 USA; 3 Beth Israel Deaconess Medical Center, 330 Brookline Ave., Boston, MA 02215 USA; 4 University of Washington, 1959 NE Pacific St., Seattle, WA 98195 USA

**Keywords:** Prostatic neoplasms, Decision making, Active surveillance, Decision aids

## Abstract

**Background:**

Although treatment decisions for localized prostate cancer (LPC) are preference-sensitive, the extent to which individuals with LPC receive preference-concordant treatment is unclear. In a sample of individuals with LPC, the purpose of this study was to (a) assess concordance between the influence of potential adverse treatment outcomes and treatment choice; (b) determine whether receipt of a decision aid predicts higher odds of concordance; and (c) identify predictors of concordance from a set of participant characteristics and influential personal factors.

**Methods:**

Participants reported the influence of potential adverse treatment outcomes and personal factors on treatment decisions at baseline. Preference-concordant treatment was defined as (a) any treatment if risk of adverse outcomes did not have *a lot* of influence, (b) active surveillance if risk of adverse outcomes had *a lot* of influence, or (c) radical prostatectomy or active surveillance if risk of adverse bowel outcomes had *a lot* of influence and risk of other adverse outcomes did not have *a lot* of influence. Data were analyzed using descriptive statistics and logistic regression.

**Results:**

Of 224 participants, 137 (61%) pursued treatment concordant with preferences related to adverse treatment outcomes. Receipt of a decision aid did not predict higher odds of concordance. Low tumor risk and age ≥ 60 years predicted higher odds of concordance, while attributing *a lot* of influence to the impact of treatment on recreation predicted lower odds of concordance.

**Conclusions:**

Risk of potential adverse treatment outcomes may not be the foremost consideration of some patients with LPC. Assessment of the relative importance of patients’ stated values and preferences is warranted in the setting of LPC treatment decision making.

*Clinical trial registration*: NCT01844999 (www.clinicaltrials.gov).

**Supplementary Information:**

The online version contains supplementary material available at 10.1186/s12911-022-01972-w.

## Background

More than 248,000 individuals will be diagnosed with prostate cancer in the United States each year, approximately 74% of whom will have clinically localized disease at the time of diagnosis [1]. Individuals who are diagnosed with clinically localized prostate cancer (LPC) may select one of several treatments, none of which are demonstrably superior in both oncologic and adverse treatment outcomes [2]. Given the preference-sensitive nature of LPC treatment decisions, the American Urologic Association (AUA) strongly recommends clinicians engage patients with LPC in shared decision making [2]. According to the AUA, shared decision making for LPC should entail patient-clinician communication about treatment options, tumor risk, and the patient’s values, preferences, life expectancy, and expected functional status [2]. Given substantial inter-individual variability in the relative importance of adverse treatment outcomes [3], AUA guidelines for the treatment of LPC stipulate that patients’ values should drive LPC treatment decisions [2]. Accordingly, at least eight decision aids for patients facing prostate cancer treatment decisions have sought to promote shared decision making by eliciting patients’ preferences and assisting patients to communicate this information to their clinicians [4]. Nevertheless, the extent to which individuals with LPC ultimately receive treatment that is concordant with their stated preferences is unclear.

One of the foremost considerations during LPC treatment decision making is the risk for adverse treatment outcomes. Potential management strategies for LPC include radical prostatectomy, external beam radiotherapy, brachytherapy, and active surveillance [2]. Compared to active surveillance, radical prostatectomy is associated with a heightened risk of urinary incontinence and sexual dysfunction, while external beam radiotherapy and brachytherapy are associated with a heightened risk of urinary obstruction, urinary irritation, sexual dysfunction, and bowel dysfunction [5]. Indeed, treatment type is the strongest predictor of urinary, sexual, and bowel quality of life six months after LPC treatment [6]. In comparison, active surveillance requires repeated physical examinations, laboratory tests, and biopsies to monitor for cancer progression. Although active surveillance is not associated with adverse physical outcomes, this management strategy may be time-consuming and has been associated with increased anxiety [7]. Given the potential impact of each management strategy on physical and psychological well-being, concordance between patients’ preferences for adverse treatment outcomes and the type of treatment they receive is an important outcome of shared decision making.

Results from studies that have assessed concordance between LPC treatment and patients’ pre-treatment preferences are mixed. In a study of 181 individuals who received a decision aid after initial consultation with a urologist, concordance between final treatment and patients’ post-intervention treatment preferences was high [8]. Conversely, in our previous multi-center trial of individuals newly diagnosed with LPC, we found that only 47% of participants who identified influential potential adverse treatment outcomes upon enrollment received treatment that was concordant with their concerns [9]. Similarly, in a study of 257 individuals who received a decision aid prior to LPC diagnosis and initial consultation with a urologist, participants’ initial treatment preferences did not predict their final treatment [10]. A fourth study found that patients with LPC who included more than one adverse bladder, bowel, or sexual treatment outcome in their list of top three concerns were more likely to receive active surveillance; however, this association was not statistically significant [11].

It is likely that the relationship between patients’ concerns about adverse treatment outcomes and final LPC treatment choice is complex. To our knowledge, no prior study has aimed to identify predictors of receiving LPC treatment that is concordant with preferences for adverse treatment outcomes. Therefore, in a sample of individuals with LPC, the purpose of this study was to assess concordance between preferences for potential adverse treatment outcomes and LPC treatment decisions. We also sought to determine whether individuals with LPC who received a decision aid would be more likely to select preference-concordant treatment than those who received usual care. Finally, we sought to identify predictors of concordance from a set of baseline demographic characteristics, clinical characteristics, personal factors, and preferences for shared decision making.

## Methods

### Study design

We conducted a prospective, multicenter, randomized controlled trial (NCT01844999) of individuals making prostate cancer treatment decisions, the details of which have been described elsewhere [12]. The primary aim of the trial was to compare the effect of the web-based, Personal Patient Profile-Prostate (P3P) decision aid on decisional conflict to that of usual care. The development of P3P [13] was guided by the Ottawa Decision Support Framework, which asserts that a high-quality decision is one that is informed and values-based [14]. The objective of the current study reflects a secondary trial aim.

### Participants

Eligible trial participants had localized, biopsy-proven cT1 or cT2 prostate cancer of any risk level; an upcoming consultation at an enrolling site; and the self-reported ability to read and understand English or Spanish. Prior to enrollment, we excluded potential participants whose records documented more than one consultation visit, a final care decision, initiation of active surveillance, or initiation of any prostate cancer treatment. Exclusion criteria were based on our experiences in the first trial of P3P, during which participants who had fewer than two consultation visits prior to enrollment derived the most benefit from the intervention [9]. We limited our analytic sample for the current study to participants with low- or favorable intermediate-risk tumors. In accordance with AUA guidelines, we defined low-risk tumors as having a Gleason score of 3 + 3 and favorable intermediate risk tumors as having a Gleason score of 3 + 4 and a prostate specific antigen level less than 10 [2]. Of these participants, we included those with complete data on the influence of potential adverse outcomes and a documented final treatment choice. We excluded participants who received treatments other than active surveillance, surgery, or radiation.

### Procedures

We recruited participants by telephone from 12 urology clinics (two of which were multidisciplinary with radiation oncology) in geographically distinct regions of the United States between September 2013 and April 2016. Following acquisition of informed consent, participants completed a baseline questionnaire on the P3P website at home or on a tablet in the clinic prior to their visit. Following baseline data collection, participants were randomized to receive the P3P decision aid plus usual education or usual care plus links to reputable websites. Six months after enrollment, research assistants prompted participants to complete follow-up questionnaires online or by mail. The study procedures were approved by the Dana-Farber Cancer Institute Institutional Review Board and the institutional review board at each recruitment site.

### Measures

#### Demographic and clinical characteristics

Participants self-reported age category, race, ethnicity, income, employment status, and educational attainment at baseline. Participants were prompted to self-report their treatment decision about six months later. We abstracted clinical tumor stage, prostate specific antigen level, and biopsy Gleason score from the medical record at baseline and verified final treatment choice in the medical record after participant self-report.

#### Influence of potential adverse treatment outcomes

Study participants rated the influence of three potential adverse outcomes of prostate cancer treatment on their treatment decision at baseline. Potential adverse treatment outcomes included bladder, bowel, and sexual dysfunction. Response options were “no influence,” “a little influence,” “some influence,” and “a lot of influence.”

#### Influence of personal factors

Study participants rated the influence of 11 personal factors on their treatment decision at baseline. Personal factors included spouse/partner, other family, friend, co-worker, famous person, “my own age,” recreation, work, perceived life expectancy, confidence in the physician, and religion. Response options were “no influence,” “a little influence,” “some influence,” and “a lot of influence.”

#### Preferred decision-making role

We assessed preferred decision-making role at baseline with the closed-ended item “please choose one statement that best says how you would like the decision about your prostate cancer care to be made.” Response options were based on preferred decision-making roles originally developed as part of the Control Preferences Scale (CPS) [15]. As in prior studies of decision role preference, we simplified the response options by collapsing the original five decision-making roles into three [6, 16]. Response options included “I prefer to make the final decision myself after thinking about my doctor’s opinion,” “I prefer that my doctor and I share the decision about which option is best,” and “I prefer that my doctor makes the final care decision, but thinks about my opinion.”

#### Concordance between influence of potential adverse outcomes and treatment choice

**Fig. 1:**
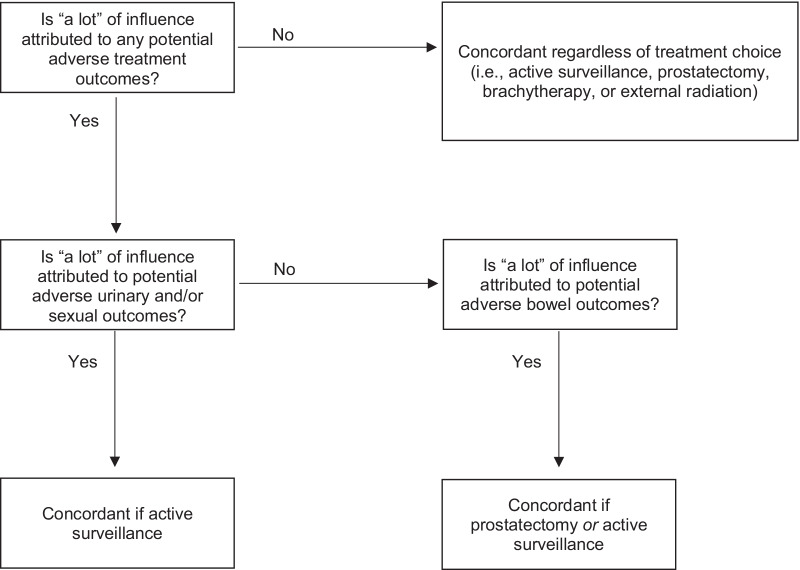
Definition of treatment concordant with influential adverse treatment outcomes

We defined concordance (Fig. 1) as selecting any active treatment or active surveillance when no potential adverse treatment outcomes had “a lot of influence.” When only adverse bowel outcomes had “a lot of influence,” we defined concordance as selecting either radical prostatectomy or active surveillance. When any other adverse treatment outcomes had “a lot of influence,” we defined concordance as selecting active surveillance.

### Analysis

We summarized participants’ demographic and clinical characteristics, influence of potential adverse treatment outcomes, preferred decision-making role, influence of personal factors, and concordance between treatment and preferences for potential adverse treatment outcomes using descriptive statistics. We used univariate logistic regression to identify potential predictors of concordance. We assessed associations between concordance and study group (decision aid vs. usual care), tumor risk (low vs. favorable intermediate), age (≥ 60 years vs. < 60 years), educational attainment (college graduate vs. not), race (Black/African-American vs. not), marital status (married/partnered vs. not), annual household income (≥ $40,000 vs. < $40,000), employment status (employed vs. not), preferred decision-making role (“I prefer to make the final decision myself after thinking about my doctor’s opinion” vs. “I prefer that my doctor and I share the decision about which option is best”/“I prefer that my doctor makes the final care decision, but thinks about my opinion”) and the influence of the 11 personal factors detailed above (“a lot of influence” vs. “no influence/a little influence/some influence”). We also conducted sensitivity analyses to identify predictors of concordance for patients with low-risk tumors, for patients with favorable intermediate risk tumors, and when the influence of personal factors was dichotomized as “a lot of/some influence” versus “a little/no influence.”

We dichotomized categorical variables to examine associations between concordance and characteristics known to be associated with the receipt of active treatment (e.g., favorable intermediate risk tumor, Black/African-American race). When categories of variables were not known to be associated with the receipt of active treatment, we dichotomized categorical variables according to the sample distribution of each characteristic. Study group and factors associated with concordance with a *p*-value < 0.25 in univariate analyses were entered into the multivariable logistic regression model. In post-hoc analyses, we used chi-square tests to compare proportions of participants undergoing specific treatments. Statistical analyses were performed in R version 3.6.2 (R Core Team, 2017) and SPSS version 24 (IBM, 2021).

## Results

### Participant characteristics

Of 392 participants who were enrolled and randomized, 63 had high-risk tumors, 71 had unfavorable intermediate risk tumors, and five were missing tumor risk data. Of the 253 participants with favorable intermediate and low-risk tumors, 21 were missing final treatment choice data and 3 underwent cryotherapy. Of the remaining 229 participants, five were missing preferences data, leaving 224 evaluable participants. Almost half (49.1%) of these participants were randomized to receive the P3P intervention. Most participants were 50–69 years old; college graduates; White, non-Hispanic; married / partnered; working; and earning ≥ $40,000 annually. Slightly more than half (50.9%) of participants had favorable intermediate risk tumors. Detailed participant demographic and clinical characteristics are provided in Table 1.

**Table 1:** Participant characteristics according to concordance between influential adverse treatment outcomes and treatment decisions

		Received concordant treatment	
	Overall	No	Yes
	(N = 224)	(N = 87)	(N = 137)
*Study group*			
Usual care	114 (50.9%)	41 (47.1%)	73 (53.3%)
Decision aid	110 (49.1%)	46 (52.9%)	64 (46.7%)
*Tumor risk and staging*			
Favorable intermediate	114 (50.9%)	60 (69.0%)	54 (39.4%)
Low	110 (49.1%)	27 (31.0%)	83 (60.6%)
Prostate specific antigen—median (IQR)	5.79 (2.84)	5.9 (2.45)	5.61 (3.01)
Gleason 3 + 3	130 (58.0%)	36 (41.4%)	94 (68.6%)
Gleason 3 + 4	94 (42.0%)	51 (58.6%)	43 (31.4%)
T1b	1 (0.4%)	0 (0.0%)	1 (0.7%)
T1c	191 (85.3%)	72 (82.8%)	119 (86.9%)
T2a	30 (13.4%)	14 (16.1%)	16 (11.7%)
T2b	2 (0.9%)	1 (1.1%)	1 (0.7%)
N0	13 (5.8%)	5 (5.7%)	8 (5.8%)
NX	211 (94.2%)	82 (94.3%)	129 (94.2%)
M0	10 (4.5%)	3 (3.4%)	7 (5.1%)
MX	214 (95.5%)	84 (96.6%)	130 (94.9%)
*Treatment choice*			
External beam radiotherapy	85 (37.9%)	19 (21.8%)	10 (7.3%)
Brachytherapy	29 (12.9%)	19 (21.8%)	10 (7.3%)
Radical prostatectomy	81 (36.2%)	49 (56.3%)	32 (23.4%)
Active surveillance	85 (37.9%)	0 (0%)	85 (62.0%)
*Age*			
≥ 70 years	36 (16.1%)	8 (9.2%)	28 (20.4%)
60–69 years	110 (49.1%)	39 (44.8%)	71 (51.8%)
50–59 years	66 (29.5%)	31 (35.6%)	35 (25.5%)
< 50 years	12 (5.4%)	9 (10.3%)	3 (2.2%)
*Education*			
Post-graduate degree	69 (30.8%)	31 (35.6%)	38 (27.7%)
Graduated college	75 (33.5%)	31 (35.6%)	44 (32.1%)
Some college	42 (18.8%)	18 (20.7%)	24 (17.5%)
Graduated high school	27 (12.1%)	6 (6.9%)	21 (15.3%)
Did not graduate high school	11 (4.9%)	1 (1.1%)	10 (7.3%)
*Race/ethnicity*			
Black/African-American	62 (27.7%)	28 (32.2%)	34 (24.8%)
White, Hispanic	10 (4.5%)	3 (3.4%)	7 (5.1%)
White, Non-Hispanic	139 (62.1%)	53 (60.9%)	86 (62.8%)
Others	13 (5.8%)	3 (3.4%)	10 (7.3%)
*Marital status*			
Married/partnered	167 (74.6%)	62 (71.3%)	105 (76.6%)
Single	22 (9.8%)	11 (12.6%)	11 (8.0%)
Divorced	28 (12.5%)	12 (13.8%)	16 (11.7%)
Separated	5 (2.2%)	2 (2.3%)	3 (2.2%)
Widowed	2 (0.9%)	0 (0%)	2 (1.5%)
*Annual household income*			
Less than $40,000	52 (23.2%)	19 (21.8%)	33 (24.1%)
$40,000 or more	153 (68.3%)	62 (71.3%)	91 (66.4%)
Missing	19 (8.5%)	6 (6.9%)	13 (9.5%)
*Work status*			
Not employed	83 (37.1%)	33 (37.9%)	50 (36.5%)
Employed	139 (62.1%)	54 (62.1%)	85 (62.0%)
Missing	2 (0.9%)	0 (0%)	2 (1.5%)

### Influence of potential adverse treatment outcomes

The influence of potential adverse treatment outcomes is detailed in Table 2. Briefly, 125/224 (55.8%) participants indicated that the potential for bladder dysfunction had “a lot of influence” on their treatment decision. Similar proportions of participants indicated that the potential for bowel dysfunction (114/224, 50.9%) and sexual dysfunction (114/224, 50.9%) had “a lot of influence” on their treatment decision. Seventy-nine of 224 participants (35.3%) reported that all three potential adverse treatment outcomes had “a lot of influence” on their treatment decision. Of these participants, 55 (69.6%) had low risk and 24 (30.4%) had favorable intermediate risk tumors. Compared to participants who did not attribute “a lot of influence” to all three potential adverse treatment outcomes, the proportions of participants in this group who underwent active surveillance, radical prostatectomy, external beam radiation, and brachytherapy were not significantly different (*p* = 0.609).

**Table 2:** Influence of potential adverse treatment outcomes and personal factors on treatment decisions by tumor risk

	Low risk	Favorable intermediate risk	Total
	(*n* = 110)	(*n* = 114)	(*n* = 224)
	*n* (%)	*n* (%)	*n* (%)
*Treatment*			
External beam radiation	7 (6.4)	22 (19.3)	29 (12.9)
Brachytherapy	4 (3.6)	25 (21.9)	29 (12.9)
Radical prostatectomy	35 (31.8)	46 (40.4)	81 (36.2)
Active surveillance	64 (58.2)	21 (18.4)	85 (37.9)
*Bladder problems*			
No influence	5 (4.5)	9 (7.9)	14 (6.3)
A little influence	12 (10.9)	12 (10.5)	24 (10.7)
Some influence	24 (21.8)	37 (32.5)	61 (27.2)
A lot of influence	69 (62.7)	56 (49.1)	125 (55.8)
*Bowel problems*			
No influence	5 (4.5)	8 (7.0)	13 (5.8)
A little influence	12 (10.9)	11 (9.6)	23 (10.3)
Some influence	30 (27.3)	44 (38.6)	74 (33)
A lot of influence	63 (57.3)	51 (44.7)	114 (50.9)
*Sexual problems*			
No influence	7 (6.4)	8 (7.0)	15 (6.7)
A little influence	19 (17.3)	17 (14.9)	36 (16.1)
Some influence	23 (20.9)	36 (31.6)	59 (26.3)
A lot of influence	61 (55.5)	53 (46.5)	114 (50.9)
*Spouse/partner*			
No influence	2 (2.4)	5 (6.1)	7 (4.2)
A little influence	15 (17.9)	17 (20.7)	32 (19.3)
Some influence	31 (36.9)	23 (28.0)	54 (32.5)
A lot of influence	36 (42.9)	37 (45.1)	73 (44.0)
*Other family*			
No influence	8 (7.3)	13 (11.5)	21 (9.4)
A little influence	36 (32.7)	42 (37.2)	78 (35.0)
Some influence	49 (44.5)	42 (37.2)	91 (40.8)
A lot of influence	17 (15.5)	16 (14.2)	33 (14.8)
*Friend*			
No influence	19 (17.4)	33 (28.9)	52 (23.3)
A little influence	43 (39.4)	47 (41.2)	90 (40.4)
Some influence	40 (36.7)	28 (24.6)	68 (30.5)
A lot of influence	7 (6.4)	6 (5.3)	13 (5.8)
*Co-worker*			
No influence	34 (30.9)	45 (40.5)	79 (35.7)
A little influence	43 (39.1)	43 (38.7)	86 (38.9)
Some influence	29 (26.4)	19 (17.1)	48 (21.7)
A lot of influence	4 (3.6)	4 (3.6)	8 (3.6)
*Famous person*			
No influence	53 (48.6)	67 (59.8)	120 (54.3)
A little influence	37 (33.9)	34 (30.4)	71 (32.1)
Some influence	14 (12.8)	9 (8.0)	23 (10.4)
A lot of influence	5 (4.6)	2 (1.8)	7 (3.2)
*My own age*			
No influence	7 (6.4)	11 (9.8)	18 (8.1)
A little influence	18 (16.5)	16 (14.3)	34 (15.4)
Some influence	47 (43.1)	41 (36.6)	88 (39.8)
A lot of influence	37 (33.9)	44 (39.3)	81 (36.7)
*Impact on recreation*			
No influence	6 (5.5)	6 (5.3)	12 (5.4)
A little influence	4 (3.7)	12 (10.5)	16 (7.2)
Some influence	31 (28.4)	40 (35.1)	71 (31.8)
A lot of influence	68 (62.4)	56 (49.1)	124 (55.6)
*Impact on work*			
No influence	19 (17.4)	13 (11.5)	32 (14.4)
A little influence	10 (9.2)	10 (8.8)	20 (9.0)
Some influence	30 (27.5)	26 (23)	56 (25.2)
A lot of influence	50 (45.9)	64 (56.6)	114 (51.4)
*Perceived life expectancy*			
No influence	5 (4.6)	6 (5.3)	11 (5.0)
A little influence	3 (2.8)	7 (6.1)	10 (4.5)
Some influence	14 (13.0)	21 (18.4)	35 (15.8)
A lot of influence	86 (79.6)	80 (70.2)	166 (74.8)
*Confidence in the physician*			
No influence	3 (2.8)	6 (5.3)	9 (4.1)
A little influence	5 (4.6)	6 (5.3)	11 (5.0)
Some influence	22 (20.4)	15 (13.3)	37 (16.7)
A lot of influence	78 (72.2)	86 (76.1)	164 (74.2)
*Religion*			
No influence	58 (52.7)	62 (54.4)	120 (53.6)
A little influence	16 (14.5)	13 (11.4)	29 (12.9)
Some influence	17 (15.5)	14 (12.3)	31 (13.8)
A lot of influence	19 (17.3)	25 (21.9)	44 (19.6)

### Influence of personal factors

The influence of personal factors is detailed in Table 2. The personal factor to which participants most often attributed “a lot of influence” was perceived life expectancy (survival). In descending order, the next most influential personal factors were confidence in the physician, impact on recreation, impact on work, “my own age,” spouse/partner, religion, other family, friend, coworker, and famous person.

### Preferred decision-making role

Of 224 participants, six (2.7%) indicated they would prefer that their physician make the final care decision, 72 (32.1%) indicated they would prefer to make the final decision themselves, and 144 (64.3%) indicated they would prefer to share the decision with their physician. Two participants (0.9%) had missing data.

### Concordance between treatment and influence of potential adverse treatment outcomes

Of 224 participants, 137 (61.2%) received treatment concordant with the influence of potential adverse treatment outcomes. Of these 137 participants, 85 (62.0%) received active surveillance and 52 (38.0%) received active treatment.

### Predictors of concordance between treatment and influence of potential adverse treatment outcomes

In univariate analyses, low tumor risk and age ≥ 60 years were significantly associated with higher odds of concordance. Conversely, attributing “a lot” of influence to perceived life expectancy, potential impact of treatment on recreation, and potential impact of treatment on work were significantly associated with lower odds of concordance (Table 3). In the multivariable model, as in univariate analyses, low tumor risk and age ≥ 60 years predicted higher odds of concordance. In terms of personal factors, attributing “a lot of influence” to the potential impact of treatment on recreation predicted lower odds of concordance. Intervention group membership was not significantly associated with concordance in either analysis.

**Table 3:** Predictors of concordance between influential adverse treatment outcomes and localized prostate cancer treatment decisions

		Univariate			Multivariable		
Variable	Category	OR	95% CI	*p*-value	OR	95% CI	*p*-value
Study group	P3P versus UC	0.8	0.5–1.3	0.369	0.9	0.4–1.8	0.681
Risk	Low versus favorable intermediate risk	3.4	2–6.1	**< 0.001**	4.9	2.2–11.8	**< 0.001**
Age	≥ 60 years versus < 60 years	2.2	1.3–3.9	**0.006**	2.5	1–6.1	**0.045**
Education	College graduate versus not	0.6	0.3–1.1	0.084	0.7	0.3–1.8	0.484
Race	B/AA versus not	0.7	0.4–1.3	0.235	0.6	0.2–1.6	0.328
Impact on recreation	“A lot” of influence versus other^a^	0.5	0.3–0.8	**0.008**	0.3	0.1–0.7	**0.005**
Impact on work	“A lot” of influence versus other^a^	0.4	0.2–0.6	**< 0.001**	0.5	0.2–1.1	0.09
Perceived life expectancy	“A lot” of influence versus other^a^	0.5	0.2–0.9	**0.03**	1.3	0.5–3.4	0.629
Spouse/Partner	“A lot” of influence versus other^a^	0.6	0.3–1.1	0.127	0.9	0.4–2	0.722
Other family	“A lot” of influence versus other^a^	0.5	0.3–1.2	0.114	0.8	0.3–2.4	0.674
My own age	“A lot” of influence versus other^a^	0.7	0.4–1.3	0.241	0.8	0.4–1.9	0.678
Marital status	Married/partnered versus not	1.3	0.7–2.4	0.368			
Income	$40,000 or more versus not	0.8	0.4–1.6	0.612			
Working status	Employed versus not	1	0.6–1.8	0.893			
Preferred decision making role	“I prefer to make the final decision about what treatment I will receive” versus other^b^	0.9	0.5–1.7	0.818			
Coworker	“A lot” of influence versus other^a^	1.1	0.3–5.3	0.933			
Friend	“A lot” of influence versus other^a^	0.7	0.2–2.3	0.588			
Famous people	“A lot” of influence versus other^a^	1.6	0.3–11.5	0.572			
Confidence in doctor	“A lot” of influence versus other^a^	0.7	0.4–1.3	0.317			
Religion	“A lot” of influence versus other^a^	1	0.5–2	0.975			

*p* < 0.05 are shown in bold

^a^“Other” includes the response options “some influence,” “a little influence,” and “no influence”

^b^“Other” includes the response options “I prefer that my doctor and I share responsibility for deciding which treatment is best for me” and “I prefer to leave all decisions regarding treatment to my doctor”

In a sensitivity analysis restricted to participants with low-risk tumors, age ≥ 60 years predicted higher odds of concordance, while being a college graduate predicted lower odds of concordance (Additional file 1). When we restricted the analysis to participants with favorable intermediate risk tumors, attributing “a lot of influence” to the impact of treatment on recreation and attributing “a lot of influence” to the impact of treatment on work were associated with lower odds of concordance (Additional file 1). In the overall sample, when we dichotomized the influence of personal factors as “a lot of/some influence” versus “a little/no influence,” having a low-risk tumor (OR = 6, 95% CI = 3.2–11.7, *p* < 0.001) and being at least 60 years old (OR = 3.2, 95% CI = 1.7–6.5, *p* = 0.001) still predicted higher odds of concordance in the multivariable analysis. However, the potential impact of treatment on recreation was no longer a significant predictor of concordance.

## Discussion

The findings of this study suggest preference for potential adverse treatment outcomes is one of several considerations that may influence LPC treatment decisions. Prior to making treatment decisions, more than half of participants attributed “a lot of influence” to the potential for bladder, bowel, or sexual dysfunction. Approximately one-third of participants attributed “a lot of influence” to all three potential adverse treatment outcomes. Nevertheless, only 61.2% of participants received treatment concordant with their preferences for potential adverse treatment outcomes.

Prior studies have identified a range of discrepancies between stated preferences and final LPC treatment decisions. In their survey of 167 individuals with newly diagnosed LPC, Sommers and colleagues [17] found that the number of years and months of life participants would be willing to trade to avoid bladder, bowel, or sexual dysfunction did not predict LPC treatment choice. In an analysis of data from the first trial of the P3P intervention [18], Bosco and colleagues [9] found that 47% of participants preferred a treatment option that was incongruent with their priority concerns. More recently, in a study of 509 individuals who completed P3P as part of clinical care, Paudel and colleagues [19] found that 67% of participants made treatment decisions that aligned with the influence of potential adverse treatment outcomes.

One possible explanation for the modest rates of preference concordance in this and other studies is that measures of patients’ preferences may be susceptible to ceiling effects [9, 20]. When preferences are assessed using a rating scale, there is no reason for respondents not to indicate that they wish to avoid a negative health outcome [21]. Likewise, rating scale responses may not provide insight into the *relative* importance of more than one negative health outcome. In our study, among participants who attributed “a lot of influence” to bladder, bowel, and sexual dysfunction, it is unclear which consideration was valued most highly. Information about the relative importance of competing considerations is needed to assess patients’ values and the extent to which they are congruent with a treatment choice [22].

Several factors may take precedence over preferences for adverse treatment outcomes. In our sample, nearly three-quarters of participants attributed “a lot of influence” to perceived life expectancy and confidence in the physician. In practice, these factors must be taken into consideration when assessing the extent to which an individual’s treatment choice is congruent with their values. For example, an individual who attributes “a lot of influence” to perceived life expectancy and the potential for adverse bladder outcomes may be risk-averse and value tumor removal above all else. While active surveillance would be concordant with this individual’s preferences for adverse treatment outcomes, it may not be the optimal choice for them overall. Indeed, in a study of 109 individuals who completed a decision aid after LPC diagnosis, longevity was the top concern of 32% of participants [8]. Likewise, in an analysis of clinical interactions between urologists and individuals with LPC, Scherr and colleagues [10] found that while urologists’ recommendations predicted treatment choice, patients’ baseline preferences did not.

The list of influential personal factors that we assessed prior to P3P administration was developed through a program of research that was grounded in the patient’s perspective [23, 24]. However, we did not assess the influence of several factors known to be associated with LPC treatment decisions. In a study of 181 individuals who completed a decision aid after LPC diagnosis, 97% of those who underwent active surveillance preferred to postpone unnecessary treatment, while 91% of those who underwent radical prostatectomy valued tumor removal [8]. In one study of 1532 individuals with LPC, greater emotional distress at the time of diagnosis and at the time of treatment decision making predicted higher odds of undergoing radical prostatectomy [25]. Similarly, a qualitative study of 20 individuals with LPC revealed treatment decisions were often driven by fear, the desire for rapid treatment, and the misconception that physical removal of the tumor would guarantee cure [26].

High-quality medical decisions occur at the intersection of patients’ values, patients’ preferences, and evidence-based recommendations [27]. In the context of LPC treatment decisions, AUA guidelines direct clinicians to “recommend” and / or “offer” certain treatments based on tumor risk [2]. These directives, which are based on evidence related to survival and quality of life outcomes, serve to define risk-concordant treatment options for patients with low- and favorable intermediate risk tumors. Risk-concordant treatment may be values-congruent for patients who prioritize survival and quality of life, but values-incongruent for those who do not. Notably, the AUA guidelines explicitly and implicitly state the need for patients’ values and preferences to inform LPC treatment decision making [2]. Concordance with AUA guidelines, then, necessarily includes the elicitation and consideration of patients’ values during a discussion of risk-concordant treatment options.

Participants’ use of the P3P decision support intervention did not predict higher odds of concordance in this sample. In this and one other multi-center randomized controlled trial, participants’ use of P3P was significantly associated with lower decisional conflict [12, 18]. Taken together, these findings highlight an important distinction between the phenomena of decisional conflict and preference concordance. Decision support tools such as P3P may reduce uncertainty and its determinants without necessarily compelling patients to select a treatment that is concordant with preferences for potential adverse treatment outcomes. Given that higher decisional conflict is associated with worse quality of life [28] and increased regret [29], it is appropriate to assess decision support interventions in terms of the extent to which they mitigate decisional conflict. However, it is important to acknowledge that interventions that reduce decisional conflict do not necessarily promote values-choice congruence [27].

In our sample, having a low-risk tumor and being at least 60 years old predicted higher odds of receiving treatment concordant with the influence of potential adverse treatment outcomes. The odds of receiving preference-concordant treatment were five times higher for patients with low-risk versus favorable intermediate risk tumors. This finding is consistent with prior research, in which individuals with LPC who preferred active surveillance over active treatment were older with fewer positive cores [7]. Given that our data were collected between September 2013 and April 2016, it is possible that the association between age, tumor risk, and receipt of preference-concordant treatment was attributable to physician recommendation. Urologists were first advised in specialty policy papers to recommend active surveillance to patients with low-risk tumors in 2017 [2]. While the use of active surveillance has increased in recent years, our findings are relevant in light of recent research that indicates urologists are reluctant to recommend active surveillance to younger patients and continue to erroneously attribute survival benefits to radical prostatectomy for patients with low-risk tumors [30]. Given that we did not assess physician recommendation in this study, it is unclear whether preference-discordant treatment decisions were driven by patients or physicians.

The results of this study suggest individuals with LPC who attribute “a lot of influence” to potential adverse treatment outcomes may contend with more than one highly influential factor when faced with a treatment decision. Clinicians may need to assist individuals with LPC to prioritize and reconcile competing values. One proposed approach to values clarification entails eliciting patients’ values and explicitly presenting the implications of those values for treatment [31]. However, limited evidence supports the use of one values clarification method over another, and few studies have explicitly assessed the extent to which values clarification exercises are associated with values-concordant decisions [31]. Clinicians should be mindful of the degree of influence their recommendation may have over the shared decision-making process. When patients’ values and preferences are not apparent, communication strategies such as agenda-setting, active listening, checking understanding, and communicating empathy may facilitate patients’ engagement in the treatment discussion [32].

Several factors limit the generalizability of our findings. First, concordance between the influence of potential adverse treatment outcomes and LPC treatment decisions may differ in samples of individuals who are consulted outside of urology clinics. Second, given the 2017 changes in AUA guidelines [2], it is possible that current patients’ and physicians’ views of LPC treatment options are not well-represented by our findings. Third, it is possible that we were underpowered to detect a statistically significant difference in concordance between categories of predictors. Reducing the number of response options on the CPS from five to three may also have affected our findings.

Our approach to defining concordance with preferences for adverse treatment outcomes was limited by several factors. First, there are cases in which an individual’s risk of experiencing an adverse treatment outcome is higher or lower than the population risk. Second, we did not measure participants’ knowledge of the risk of adverse treatment outcomes and cannot evaluate the extent to which participants had an accurate understanding of these risks during treatment decision making. Finally, as discussed above, it is possible that participants’ treatment decisions were influenced by a factor that was not assessed in this study.

## Conclusions

As the results of this and other studies make clear, patient preferences related to potential adverse treatment outcomes may not align with LPC treatment choice. It is possible patients value other factors more highly than the potential for adverse treatment outcomes during LPC treatment decision making. Future studies that evaluate decision support interventions should evaluate the relative importance of multiple factors, and research to identify associations between values-concordant choices and health outcomes is warranted.

## Supplementary Information

**Additional file 1**. Predictors of concordance between the influence of potential adverse treatment outcomes and localized prostate cancer treatment decisions.

## Data Availability

The datasets analyzed during the current study are not publicly available because they contain protected health information. The data that support the findings of this study are available from the authors on reasonable request.

## Abbreviations

LPC: Localized prostate cancer
AUA: American Urologic Association
P3P: Personal Patient Profile-Prostate
CPS: Control Preferences Scale
