# Supplementary material for: Concordance between influential adverse treatment outcomes and localized prostate cancer treatment decisions
Source: BMC Med Inform Decis Mak. 2022 Aug 24;22:223. doi: 10.1186/s12911-022-01972-w (PMC9404592; doi:10.1186/s12911-022-01972-w)
Supplement: Supplementary file 1 — Additional file 1. Predictors of concordance between the influence of potential adverse treatment outcomes and localized prostate cancer treatment decisions. [file 12911_2022_1972_MOESM1_ESM.doc]

**Supplement 1.** Predictors of concordance between the influence of potential adverse treatment outcomes and localized prostate cancer treatment decisions

|  |  | **All Participants** | | | | | | **Participants with  Low Risk Tumors** | | | | | | **Participants with  Favorable Intermediate Risk Tumors** | | | | | |  |  |  |  |  |  |
| --- | --- | --- | --- | --- | --- | --- | --- | --- | --- | --- | --- | --- | --- | --- | --- | --- | --- | --- | --- | --- | --- | --- | --- | --- | --- |
|  |  | **Univariate** | | | **Multivariable** | | | **Univariate** | | | **Multivariable** | | | **Univariate** | | | **Multivariable** | | |  |  |  |  |  |  |
| **Predictors** | | **OR** | **95% CI** | ***p*** | **OR** | **95% CI** | ***p*** | **OR** | **95% CI** | ***p*** | **OR** | **95% CI** | ***p*** | **OR** | **95% CI** | ***p*** | **OR** | **95% CI** | ***p*** |  |  |  |  |  |  |
| Study group | | | | | | | | | | | | | | | | | | | |  |  |  |  |  |  |
|  | Decision aid vs. usual care | 0.8 | 0.5-1.3 | 0.369 | 0.9 | 0.4-1.8 | 0.681 | 2 | 0.8-5 | 0.149 | 2.7 | 1-8.6 | 0.066 | 0.5 | 0.2-1 | **0.043** | 0.4 | 0.1-1.2 | 0.121 |  |  |  |  |  |  |
| Demographic and clinical characteristics | | | | | | | | | | | | | | | | | | | |  |  |  |  |  |  |
|  | Low vs. favorable intermediate risk | 3.4 | 2.0-6.1 | **<0.001** | 4.9 | 2.2-11.8 | **<0.001** | *Not applicable* | | | | | | *Not applicable* | | | | | |  |  |  |  |  |  |
|  | Age ≥60 years vs.<60 years | 2.2 | 1.3-3.9 | **0.006** | 2.5 | 1.0-6.1 | **0.045** | 3.8 | 1.6-9.6 | **0.004** | 5.1 | 1.7-17.1 | **0.005** | 1.7 | 0.8-3.9 | 0.171 | 1.4 | 0.4-5.3 | 0.585 |  |  |  |  |  |  |
|  | College graduate vs. not | 0.6 | 0.3-1.1 | 0.084 | 0.7 | 0.3-1.8 | 0.484 | 0.5 | 0.2-1.3 | 0.198 | 0.2 | 0.1-0.8 | **0.024** | 0.6 | 0.3-1.3 | 0.232 | 1.4 | 0.4-5 | 0.57 |  |  |  |  |  |  |
|  | Black / African-American vs. not | 0.7 | 0.4-1.3 | 0.235 | 0.6 | 0.2-1.6 | 0.328 | 0.5 | 0.2-1.3 | 0.132 | 0.5 | 0.1-1.5 | 0.19 | 1 | 0.4-2.1 | 0.919 | - | - | - |  |  |  |  |  |  |
|  | Married/Partnered vs. not | 1.3 | 0.7-2.4 | 0.368 | - | - | - | 1.2 | 0.4-3.1 | 0.747 | - | - | - | 1.4 | 0.6-3.2 | 0.478 | - | - | - |  |  |  |  |  |  |
|  | Annual household income ≥$40,000 vs. not | 0.8 | 0.4-1.6 | 0.612 | - | - | - | 1.3 | 0.5-3.5 | 0.565 | - | - | - | 0.6 | 0.3-1.6 | 0.342 | - | - | - |  |  |  |  |  |  |
|  | Employed vs. not | 1.0 | 0.6-1.8 | 0.893 | - | - | - | 1.1 | 0.4-2.5 | 0.91 | - | - | - | 1.2 | 0.5-2.5 | 0.71 | - | - | - |  |  |  |  |  |  |
| Influence attributed to personal factors | | | | | | | | | | | | | | | | | | | |  |  |  |  |  |  |
|  | Impact on recreation (“a lot” vs. other ^a^) | 0.5 | 0.3-0.8 | **0.008** | 0.3 | 0.1-0.7 | **0.005** | 0.5 | 0.2-1.3 | 0.153 | 0.5 | 0.1-1.5 | 0.228 | 0.3 | 0.1-0.6 | **0.002** | 0.2 | 0.1-0.6 | **0.006** |  |  |  |  |  |  |
|  | Impact on work  (“a lot” vs. other ^a^) | 0.4 | 0.2-0.6 | **<0.001** | 0.5 | 0.2-1.1 | 0.09 | 0.5 | 0.2-1.3 | 0.169 | 1.1 | 0.4-3.5 | 0.872 | 0.3 | 0.1-0.6 | **0.001** | 0.3 | 0.1-1 | **0.047** |  |  |  |  |  |  |
|  | Perceived life expectancy  (“a lot” vs. other ^a^) | 0.5 | 0.2-0.9 | **0.030** | 1.3 | 0.5-3.4 | 0.629 | 0.6 | 0.2-1.8 | 0.411 | - | - | - | 0.3 | 0.1-0.7 | **0.006** | 0.5 | 0.1-2.1 | 0.381 |  |  |  |  |  |  |
|  | Spouse/Partner  (“a lot” vs. other ^a^) | 0.6 | 0.3-1.1 | 0.127 | 0.9 | 0.4-2.0 | 0.722 | 0.7 | 0.2-1.9 | 0.461 | - | - | - | 0.5 | 0.2-1.3 | 0.178 | 0.9 | 0.3-3 | 0.851 |  |  |  |  |  |  |
|  | Other family  (“a lot” vs. other ^a^) | 0.5 | 0.3-1.2 | 0.114 | 0.8 | 0.3-2.4 | 0.674 | 0.4 | 0.1-1.2 | 0.09 | 0.6 | 0.2-2.5 | 0.477 | 0.6 | 0.2-1.9 | 0.418 | - | - | - |  |  |  |  |  |  |
|  | My own age  (“a lot” vs. other ^a^) | 0.7 | 0.4-1.3 | 0.241 | 0.8 | 0.4-1.9 | 0.678 | 0.7 | 0.3-1.7 | 0.391 | - | - | - | 0.8 | 0.4-1.7 | 0.58 | - | - | - |  |  |  |  |  |  |
|  | Coworker  (“a lot” vs. other ^a^) | 1.1 | 0.3-5.3 | 0.933 | - | - | - | 1 | 0.1-20.2 | 0.983 | - | - | - | 1.1 | 0.1-9.8 | 0.898 | - | - | - |  |  |  |  |  |  |
|  | Friend  (“a lot” vs. other ^a^) | 0.7 | 0.2-2.3 | 0.588 | - | - | - | 0.4 | 0.1-2.2 | 0.265 | - | - | - | 1.1 | 0.2-6.3 | 0.895 | - | - | - |  |  |  |  |  |  |
|  | Famous people  (“a lot” vs. other ^a^) | 1.6 | 0.3-11.5 | 0.572 | - | - | - | 0.5 | 0.1-3.8 | 0.429 | - | - | - | *Not applicable; insufficient variation in responses* | | | | | |  |  |  |  |  |  |
|  | Confidence in doctor  (“a lot” vs. other ^a^) | 0.7 | 0.4-1.3 | 0.317 | - | - | - | 0.7 | 0.2-1.9 | 0.54 | - | - | - | 0.8 | 0.3-1.8 | 0.555 | - | - | - |  |  |  |  |  |  |
|  | Religion  (“a lot” vs. other ^a^) | 1.0 | 0.5-2.0 | 0.975 | - | - | - | 0.9 | 0.3-3 | 0.844 | - | - | - | 1.3 | 0.5-3.1 | 0.6 | - | - | - |  |  |  |  |  |  |
| Decision role preference | | | | | | | | | | | | | | | | | | | |  |  |  |  |  |  |
|  | “I prefer to make the final decision about what treatment I will receive” vs. other^b^ | 0.9 | 0.5-1.7 | 0.818 | - | - | - | 1.2 | 0.5-3 | 0.685 | - | - | - | 0.4 | 0.2-1.1 | 0.075 | 0.1 | 0-0.5 | **0.006** |  |  |  |  |  |  |

^a^ “Other” includes the response options “some influence,” “a little influence,” and “no influence.”

^b^ “Other” includes the response options “I prefer that my doctor and I share responsibility for deciding which treatment is best for me” and “I prefer to leave all decisions regarding treatment to my doctor.”
